# An audit of referral patterns for glioblastoma patients in Beaumont hospital

**DOI:** 10.1186/1753-6561-6-S4-O48

**Published:** 2012-07-09

**Authors:** J Clerkin

**Affiliations:** 1 Royal College of Surgeons in Ireland

## Introduction

This audit examined the referral patterns for newly diagnosed glioblastoma patients at a specialised tertiary referral centre, to uncover any intrinsic delays within the system. Differences in the interval for admission and surgery in relation to different referral sources and symptomology were analysed. Such delays negatively impact on patient care.

## Methods

Medical case notes for 81 histologically confirmed GBM patients who underwent surgery in Beaumont hospital in 2010 were examined. Information regarding patient demographics, referral source, symptomology and dates of first presentation, CT scanning, admission and surgery were extracted.

## Results

The median time from CT diagnosis to admission was 6 days, and 3.3 days for subsequent surgery. Variations existed between referral sources, with those presenting to A+E with neurological deficits being diagnosed more promptly.

## Conclusions

Newly diagnosed glioblastoma patients experience delays in accessing neurosurgical care with location of first presentation and severity of symptomology being the most influential factors.

